# Hemophilia A with a Rare Presentation of Hemarthrosis and Arthropathy Involving Multiple Joints in a Young Male Child

**DOI:** 10.7759/cureus.4524

**Published:** 2019-04-23

**Authors:** Zainab Majid, Faryal Tahir, Laila Tul Qadar, Mahnoor Y Shaikh, Sayed Mustafa Mahmood Shah

**Affiliations:** 1 Internal Medicine, Dow University of Health Sciences, Karachi, PAK

**Keywords:** hemophilia, arthropathy, factor viii, hemarthrosis, coagulaopathy, inherited disorders, hemophilic arthropathy, hemophilia a

## Abstract

Hemophilia A is an X-linked hereditary bleeding disorder that is rarely encountered by most physicians and surgeons in their practice. Patients with mild hemophilia A tend to bleed profusely after surgery or trauma whereas a severe variant may manifest as spontaneous bleeding after minor trauma, mainly into the joints and muscles. However, seldom do we find a case where the patient experiences bleeding into multiple joints at the same time. In the South Asian population, the incidence of hemarthrosis in hemophilic patients holds scarce literature, making this an under-reported entity.

## Introduction

Hemophilia A is a rare X-linked hereditary bleeding disorder due to the deficiency of coagulation factor VIII (FVIII) [1]. It is most commonly seen in males, as they only have one X chromosome. Depending on the FVIII activity in patient's plasma, hemophilia A is classified as severe (<1%), moderate (1%-5%), or mild (>5% to <40%) [2]. Patients with mild hemophilia A tend to bleed profusely after minor surgery or trauma. On the other hand, patients with severe hemophilia A suffer from frequent episodes of spontaneous bleeding after minor trauma, mainly into the joints and muscles. Repeated episodes of hemarthrosis can lead to arthropathy. It is postulated that the chronic toxic effects of blood and inflamed synovium lead to the erosion of the cartilage and subchondral bone. Severe hemophilic arthropathy (HA) is seen in 50% of cases [3]. However, rarely do we find a case where the patient experiences bleeding into multiple joints at the same time especially in an underdeveloped country, such as Pakistan, where blood coagulation disorders, such as hemophilia A, are under-reported.

## Case presentation

A 12-year old boy, known case of hemophilia A, was brought to the emergency department (ED) of Dr. Ruth KM Pfau, Civil Hospital Karachi (CHK) in February 2019 with the complaint of swelling and pain in multiple joints along with intermittent fever for 13 days. Swelling initiated from the left elbow joint followed by a sudden, dull, aching pain, exacerbated by activity and associated with a limited range of movement. Seven days later, it was followed by a similar joint ache and swelling in the right elbow joint, then in the left knee joint, and, lastly, in the right shoulder joint. There was no history of any trauma to the joint. The patient also experienced high-grade fever, intermittent in nature, with severe joint ache, which was relieved by taking oral antipyretics.

Our patient had experienced similar episodes of joint ache occasionally since birth, and all of them were relieved by the injection of FVIII and transfusion of blood. He also had a history of occasional non-traumatic episodes of epistaxis and skin bruising since birth. Past surgical history revealed a difficulty in blood clotting after circumcision, which required medical management. The patient's vaccinations were up to date according to the expanded program of immunization (EPI). He is the first product of a consanguineous marriage, where the second product is a seven-year-old male with similar complaints as to our patient.

**Figure 1:**
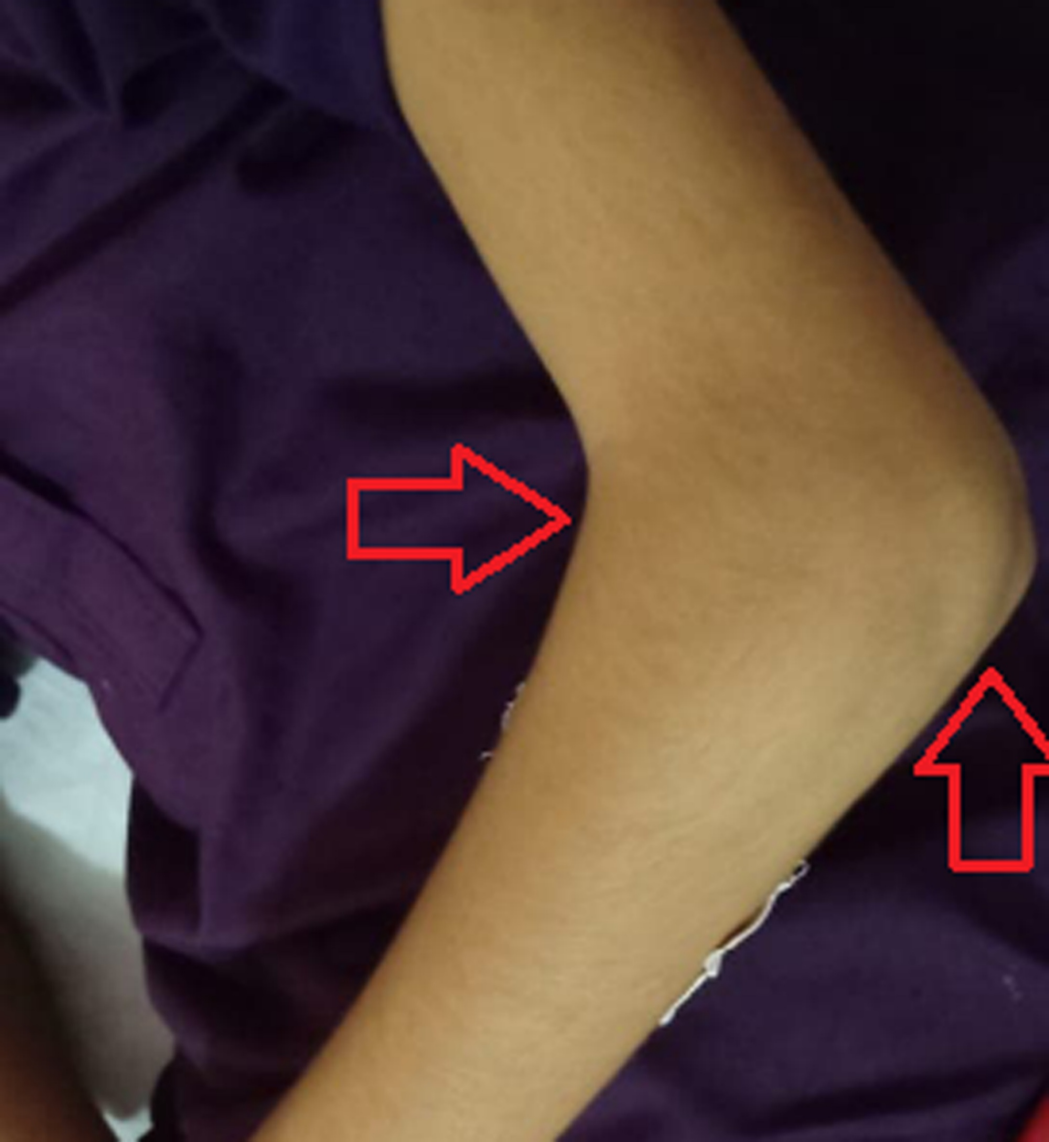
Right elbow showing mild swelling

**Figure 2:**
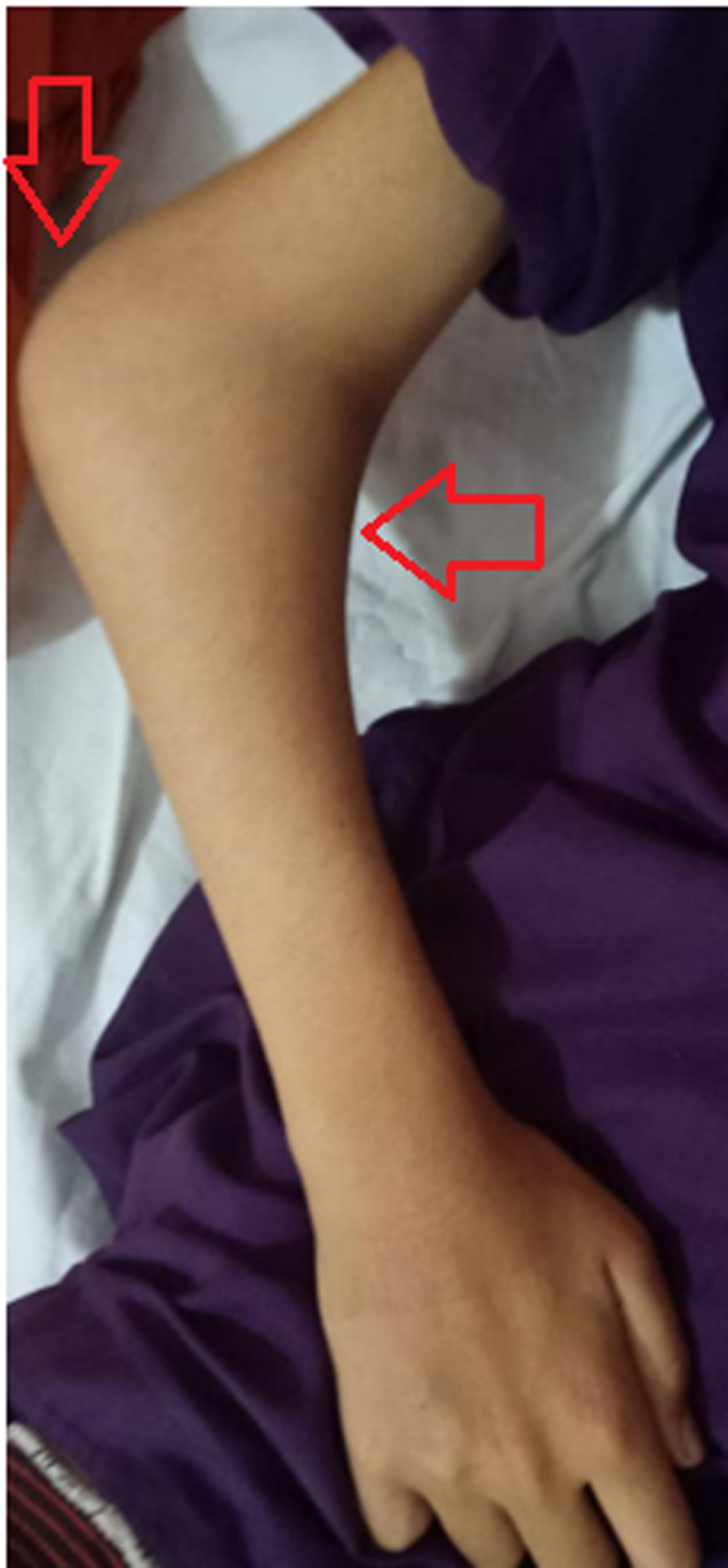
Left elbow in semi-flexed position showing large swelling

**Figure 3:**
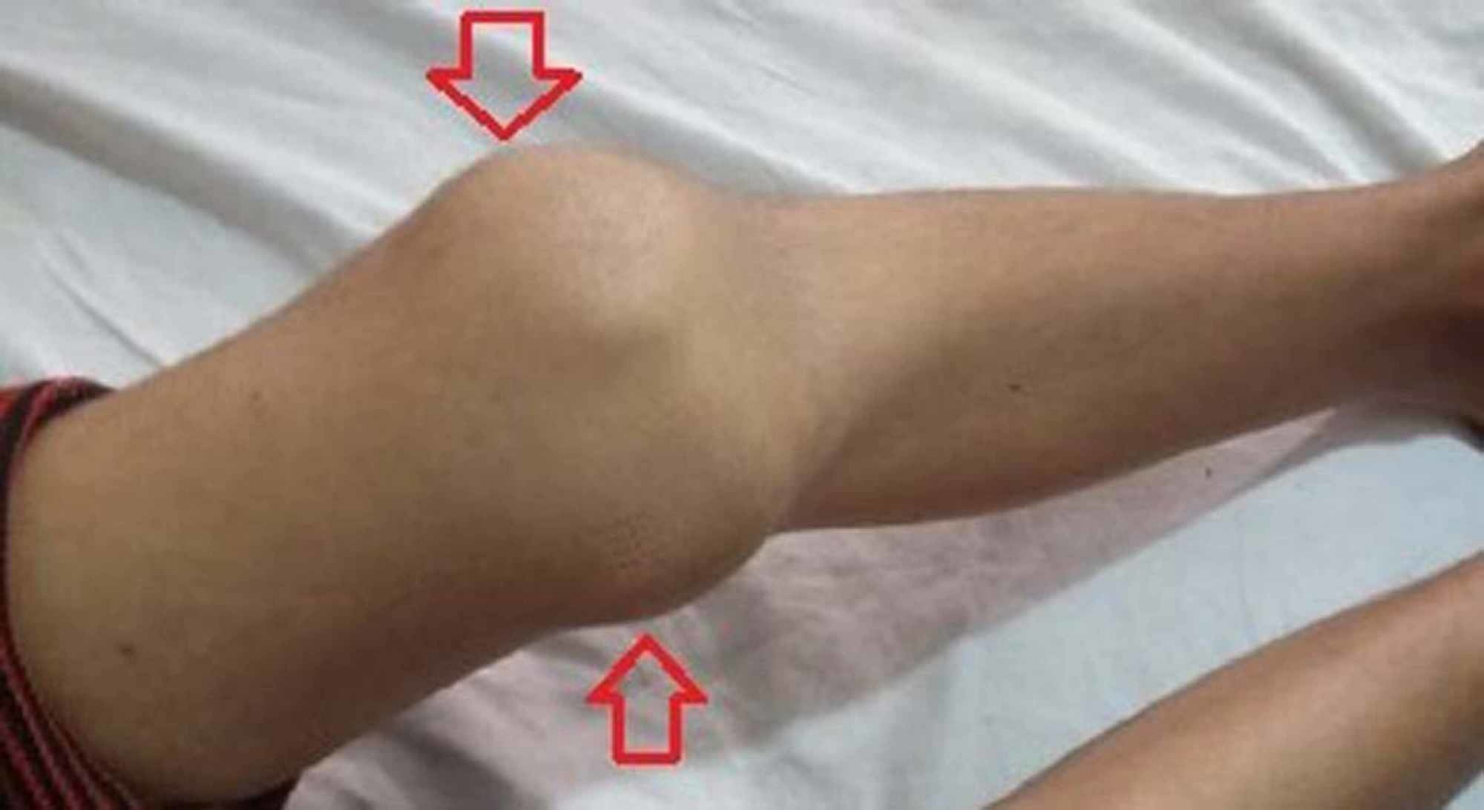
Left knee flexed at 90 degrees with localized swelling

On examination (O/E), a young boy of average height and lean built was found oriented to time, place, and person with a Glasgow coma scale (GCS) of 15/15. His heart rate (HR) was 88 beats/min, blood pressure (BP) was 110/80 mmHg, respiratory rate (RR) was 20 breaths/min, and he was febrile (102°F), with no visible bruises. Upon locomotor examination (Table 1), swelling with or without tenderness and restricted range of movement in the involved joints was noted (Figures 1-3). All other systems were found unremarkable.

**Table 1:** Findings of locomotor examination

JOINT INVOLVED	SWELLING AND TENDERNESS	RANGE OF MOVEMENT
Right elbow	A swelling (5x5 cm) limited to the joint. Warm and tender on palpation.	Flexion up to 45 degrees, extension up to 150 degrees.
Left elbow	Large swelling (10x10 cm) extending from mid-arm to forearm. Warm and tender on palpation.	Kept in semi-flexed position.
Right shoulder	No obvious swelling or tenderness.	Limited to 90 degrees of abduction.
Left knee	Local swelling of joint which was not tender.	Fixed in 90 degrees of flexion.

Upon history and physical examination, a diagnosis of hemophilia A with hemarthrosis and HA was suspected for which two pints of fresh frozen plasma (FFP) were transfused until FVIII was arranged. Injection Provas was given to relieve pain. Laboratory (lab) investigations were sent. Upon stabilization, the patient was transferred to the ward.

Lab investigations revealed hemoglobin (Hb) of 7.5 gm/dl, mean corpuscular volume (MCV) of 82 fl (Normal (N) = 76-96), mean corpuscular hemoglobin concentration (MCHC) of 33.2 gm/dl (N = 32-36), total leukocyte count (TLC) of 8.7 x 103/μL (N = 4-11), and platelet count (PLT) of 309 x 103/μL (N = 150-400). The level of C-reactive protein (CRP) was found to be 30.7 mg/L (N = <5). The clotting profile showed an international normalized ratio (INR) of 1.04, prothrombin time (PT) of 10.9 seconds and activated partial thromboplastin time (aPTT) of 29 seconds. FVIII levels were found to be less than 1%.

A radiologic examination of the right elbow, left elbow, and left knee showed Grades 1, 2, and 5 arthropathic changes, respectively, upon X-rays of the anteroposterior (AP) and lateral views. Therefore, a final diagnosis of severe hemophilia A with hemarthrosis and HA was made. FVIII was arranged and injected 1750 international units (IU) intravenous (I/V) on Day 1 followed by 700 IU I/V on Days 2, 3, and 5 with subsequent improvement in the patient’s condition. The further plan was to give FVIII on every alternate day till symptoms completely resolved.

The patient was counseled for regular follow-up after every three to four weeks, FVIII prophylaxis and at least 60 minutes of low-impact daily physical activity. He was also taught to immediately report to the emergency department (ED) in case of any joint swelling and pain, bruising, epistaxis, blood in urine or stool, severe headache, or weakness on any side of the body.

## Discussion

Hemophilia A and B are rare, life-threatening, inherited coagulation disorders with one of the main complications being HA, which results from bleeding into the joints. Approximately half of the hemophilic population is estimated to develop HA at some point in their life span [4]. Even though the initial presentation of hemophilia consists of bleeding after minor falls and trauma, particularly after circumcision at birth, repeated mucosal bleeding, and easy bruising, HA remains the characteristic manifestation of this disease [5].

The exact mechanism of HA leading to the development of arthropathy is unclear, although the probable pathogenesis has been suggested by some studies. A multiprotein complex called "inflammasome," which causes the secretion of interleukin (IL)-1β has been found to play a major role. Moreover, the importance of iron in the regulation of certain pro-inflammatory cytokines like IL-1α, IL-6, and tumor necrosis factor (TNF)-α has also been highlighted [6]. The joints that are more prone to this phenomenon of arthropathy include the knees, elbows. and ankles [5], however, the less common association of the shoulder joint was also observed in our patient. Contrary to the usual manifestation of hemarthrosis, i.e. single joint damage at one time [4], our patient presented with the simultaneous involvement of the bilateral elbow, left knee, and shoulder joint, which is not commonly seen.

Hemarthrosis has different clinical presentations, depending on the age of the hemophilic patient. Younger children exhibit irritability and failure to demonstrate movement with the affected joint whereas symptoms in adolescents and young adults include aching associated with tingling, stiffness, and swelling [4]. In our case, marked restriction in movement was the chief complaint apart from prominent swelling and pain. Since our patient also presented with the complaint of fever along with the involvement of multiple joints, the possible differential of juvenile rheumatoid arthritis (RA) could be misleading in making a diagnosis in other similar cases. 

Recurrent episodes of bleeding into the joint, either spontaneous or following trauma, hamper the normal homeostatic balance of chondrocytes, leading to the loss of joint space along with cystic changes, muscle degeneration, and osteoporosis [7-8]. These events, thus, contribute to the development of HA. Use of plain radiographs for the detection and evaluation of HA has shown consistent results, however, recent studies have supported the use of magnetic resonance imaging (MRI) for early diagnosis and, concurrently, early intervention for improved outcomes [9].

Acute hemarthrosis is initially managed with ice, cold compressions, and analgesics for minimizing the pain [4]. The gold standard management for acute bleeds in severe hemophilia consist of factor replacement therapy [8] and, as in our case, the transfusion of FVIII. With proper treatment, symptoms undergo complete resolution within a few weeks [4] as observed in our patient. In developed parts of the world, hemophilia A is managed with routine prophylaxis with the administration of factors on every alternate day, thus reflecting on their reduced incidence of HA in hemophilics [8].

## Conclusions

Rare cases of hemophilia A presenting with hemarthrosis and subsequent HA involving multiple joints should be timely diagnosed by physicians and surgeons. Using plain radiographs, or preferably MRI, and some blood tests, early diagnosis of this unusual entity can prevent its progression to severity. If managed properly, this disease carries favorable prognosis. Also, physicians should counsel their patients to carry out routine prophylaxis and physical activity.
